# A novel approach for deoxycholic acid administration to treat submental fullness: A case report assessed by 3D stereophotogrammetry

**DOI:** 10.1016/j.jobcr.2024.03.011

**Published:** 2024-03-29

**Authors:** Victor Rogerio, Viviane Rabelo, Pietra Roschel, Tatiane Sakemi, Marcelo Germani, Victor R.M. Munoz-Lora

**Affiliations:** a Let's HOF Academy, São Paulo, Brazil; b Department of Periodontology and Implantology, Guarulhos University, São Paulo, Brazil

**Keywords:** Deoxycholic acid, Submental fat, Lipolysis, Stereophotogrammetry

## Abstract

**Background:**

While effective, DAc injections for submental fat (SMF) reduction carry risks, including vascular damage and skin necrosis when improperly administered. This study presents a novel approach to SMF reduction using blunt microcannulas for DAc injections, coupled with 3D stereophotogrammetry quantification (3D-SQ).

**Clinical presentation:**

A 47-year-old female with SMF underwent two DAc applications. 3D-SQ was performed before and after each treatment using a 3D-SQ system. The patient experienced a substantial total volume reduction of 14.81 mL in the submental area after two DAc applications. 3D-SQ analysis showed a gradual reduction in submental volume over time. Importantly, no serious adverse events were reported, with only minor pain and warmth at the treated site. The reduction of SMF through DAc injections involves adipocyte cell lysis, emphasizing the importance of proper injection technique to avoid adverse events. The use of blunt microcannulas offers a safer alternative, minimizing the risk of skin necrosis, ulceration, and intra-arterial injections. Additionally, cannulas reduce bruising due to their blunt design and fan technique, enhancing patient comfort and safety.

**Conclusion:**

This case report highlights the efficacy of a novel cannula approach for DAc SMF reduction, assessed by 3D-SQ. Blunt microcannulas may represent a safer option compared to hypodermic needles, reducing the likelihood of severe complications.

## Graphical abstract

A) A cannula approach for deoxycholic acid administration to treat submental fullness. B) Volume decrease and C) height difference at 90 days from baseline.

**Figure:**
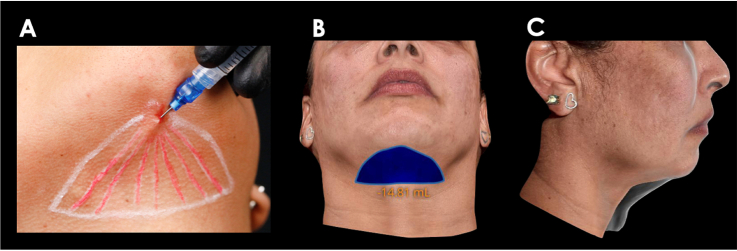

## 1 Introduction

The attractiveness of a person's face can be directly linked to the harmonious balance between their facial features and neck. Enzymatic submental fat (SMF) reduction is a minimally invasive cosmetic procedure designed to eliminate fat and reduce submandibular volume, ultimately enhancing the overall contour and the transition between the head and neck in a non-invasive manner.^1^, ^2^, ^3^, ^4^

SMF lipolysis involves the application of ATX-101, a synthetic compound structurally identical to the body's own deoxycholic acid (DAc). ATX-101 acts like a detergent, breaking down the cell membranes of adipocytes (fat cells), leading to the necrosis of these fat cells and a reduction in volume in the treated area.^5^ A retrospective study, which examined 56,320 lipolytic injections, has demonstrated the safety and effectiveness of this procedure.^6^

However, it's crucial to note that incorrect administration of DAc can result in severe adverse events, such as vascular damage, skin necrosis, among others.^3^^,^^7^ These events can occur when the product is injected into unintended areas due to improper technique or the incorrect placement of needles in inappropriate depths, either too superficially or too deeply, causing the product to spread into the skin or muscles.

On the contrary, blunt microcannulas are commonly used devices for injecting fillers into subdermal layers. They tend to cause less bruising, ecchymosis (skin discoloration), and pain compared to traditional hypodermic needles. The following report will demonstrate the effectiveness of applying DAc for SMF lipolysis using blunt microcannulas, with an assessment conducted using 3D stereophotogrammetry quantification (3D-SQ).

## 2 Case presentation

A 47-year-old female patient visited the Let's HOF clinic in São Paulo, Brazil, with concerns about submental fat (SMF) and a lack of definition in her lower facial contour. The patient had not undergone any prior treatments in the submandibular region. Following a comprehensive evaluation by our expert team, it was determined that there was no skin sagging, but SMF was indeed present. Consequently, a lipolytic treatment with two DAc sessions was recommended.

## 3 Deoxycholic acid application

**Fig. 1:**
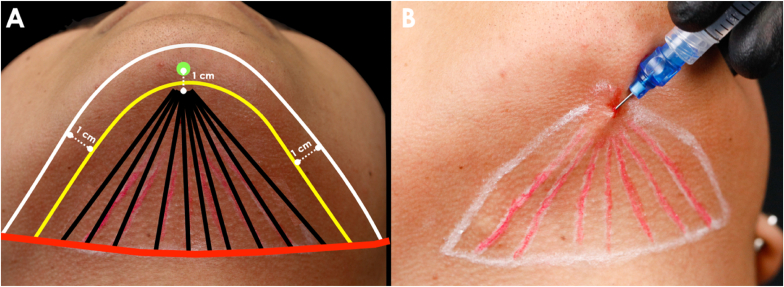
Schematic illustration of the A) marking and B) application. White line: lower border of the mandible; yellow line: anterior boundary of the treated area; red line: posterior boundary of the treated region (hyoid bone); green dot: entry point for the cannula; black lines: retroinjections pathways.

After cleansing the face and neck with soap and water, the submental area was meticulously prepared with aseptic techniques using 2% chlorhexidine. Subsequently, we proceeded to outline the submental fat (SMF) compartment, guided by key external anatomical landmarks (Fig. 1A). To define the anterior boundary, a line was drawn 1 cm away from the inferior mandibular border, ensuring safe distance from the marginal mandibular nerve. The posterior boundary was identified at the level of the hyoid bone.^8^^,^^9^

Once the injection site was established, we created an entry point for the cannula using a 22G needle (Terumo, Brazil). The entry point was located 1 cm away from the uppermost edge of the anterior boundary line to ensure that the DAc injection was not administered too superficially. Topical anesthesia (Pliaglis, Galderma, Brazil) was applied to minimize discomfort from needle insertion. To administer the product, we connected a 5 mL luer-lock syringe (BD – Becton Dickson) to a 22G-50 mm microcannula (Smart Cânula, SmartGR, Brazil). We then evenly distributed 0.5 mL of DAc (Phosphocol, Toskani, Spain) with a total of 10 retroinjections within the demarcated submental fat compartment, amounting to 5 mL of the product (Fig. 1B). We concluded the procedure with a gentle massage of the treated area and provided the patient with post-treatment instructions. The entire process was carried out by a highly experienced injector. Two DAc applications were performed, spaced 45 days apart as part of the treatment protocol.

### 3.1 3D stereophotogrammetry assessment (3D-SQ)

We conducted a facial scan using a 3D-SQ system (3D Life Viz™ Mini-Quantificare, Sophia Antipolis, France) at three different time points: baseline, 45, and 90 days after the treatment. The 3D-SQ system utilizes passive stereophotogrammetry and incorporates a specialized digital camera equipped with 13.5–24 million pixels (Nikon 3200, Nikon, Japan). During the process, photographs were taken from a standardized distance of 70 cm, and the camera was held perfectly perpendicular to the target point.

The 3D-SQ camera simultaneously captures two images from distinct angles, and a proprietary software (DermaPix Database for photo documentation, Quantificare, Sophia Antipolis, France) automatically combines these stereo images to generate a three-dimensional reconstruction. The LifeViz™ application, employed for processing and managing 3D images, calculates and identifies variations between the examined images.^10^ This software also offers specific tools for comparing the 3D images acquired before and after the procedures. It can precisely record soft tissue displacement and compute volumetric changes in the specified area by estimating the observed differences. These alterations are then quantified in milliliters (mL) and presented on a color-coded scale, with regions of volume gain depicted in yellow and areas of volume loss highlighted in green. This representation effectively conveys the achieved results.

## 4 Results

**Fig. 2:**
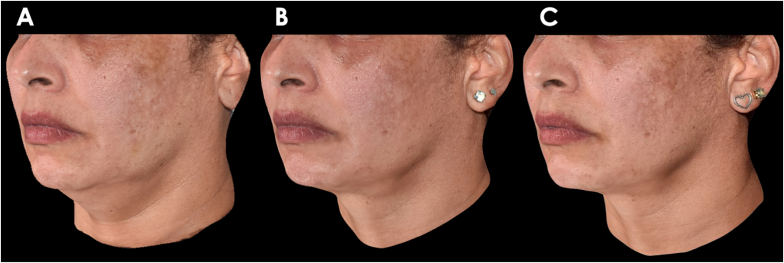
Assessment of the patient at A) baseline, B) 45 days after treatment and C) 90 days after treatment. Photos authorized by the patient.

**Fig. 3:**
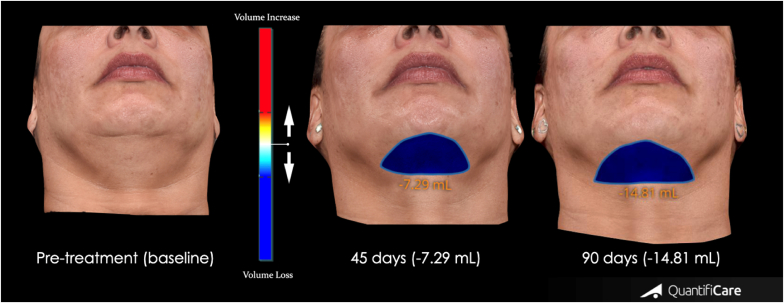
Volumetric changes measured over 90 days using a 3D stereophotogrammetry device (3D Life Viz™ Mini-Quantificare, Sophia Antipolis, France). Photos published with patient's consent. The shades between blue and red represent gains or losses in volume. Red: volume gain; Blue: volume loss.

**Fig. 4:**
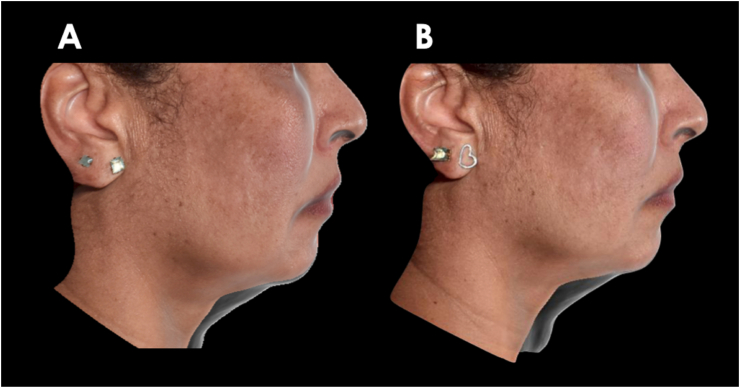
Height difference at A) 45 days and B) 90 days from baseline. Photos published with patient's consent.

The 3D-SQ analysis consistently demonstrated a gradual reduction in the submental area following each DAc treatment (Fig. 2). The initial application of DAc led to a significant total volume reduction of 7.29 mL within the submental region. Subsequently, after the second DAc treatment, we observed a further volume decrease of 7.52 mL, resulting in a cumulative submental volume reduction of 14.81 mL compared to the baseline (pre-treatment) measurements (Fig. 3). A remarkable height reduction of the submandibular area at 45 and 90 days when compared to baseline measurement was observed (Fig. 4).

Importantly, no serious adverse events were reported by the patient. The patient did, however, experience low to moderate pain, which spontaneously resolved without the need for any specific treatment. Additionally, the patient noted a sensation of warmth in the treated region.

## 5 Discussion

This case report introduces an innovative approach for administering DAc in the submental region to treat SMF. Following two DAc applications using blunt microcannulas, our patient achieved a remarkable total volume reduction of 14.81 mL. This reduction occurred gradually over time and was quantified using 3D-SQ, with only mild and transient adverse events reported.

The reduction in SMF is attributed to the reduction of adipocytes through cell lysis caused by DAc application. DAc is injected into the subcutaneous fat layer located between the dermis and the platysma muscle.^9^ Typically, applications of DAc involve using a 30G 0.5-inch needle attached to a syringe to distribute 2 mg/cm^3^ of the product into the subcutaneous fat. The number of treatments for patients depends on the results and is performed 28 days apart, with the total not exceeding 6 treatments per patient.^1^^,^^2^ It's essential to consider factors such as injection pressure and volume, but the variable needle-depth appears to play a critical role in preventing adverse events and ensuring successful outcomes.^9^

Common adverse events, including local pain, bruising, edema, and numbness, are transient and self-resolving. However, more severe adverse events have been reported previously.^11^ Superficial injections can lead to DAc deposition near the dermis, potentially resulting in ulceration or skin necrosis.^12^ Alopecia has also been reported as a side effect, though its mechanism remains unclear. Conversely, overly deep injections or improper needle placement may lead to DAc deposition in the platysma muscle or the postplatysmal SMF. Also, intra-arterial injections of DAc, causing skin necrosis, have also been documented.^13^

All these adverse events can be mitigated by employing cannulas for DAc application in SMF. Cannulas, which have blunt ends, are commonly used to deliver hyaluronic acid fillers into subcutaneous fat pads. Unlike needles, cannulas are considered safer as they cannot puncture tissue. Moreover, they are often used with a fan technique, which can reduce the incidence of bruising caused by needles and ensure even product distribution in the region. On the other hand, the use of cannulas may require a higher level of expertise from the injector to maintain a consistent product extrusion and distribute it evenly in the desired area.

To best of our knowledge, this is the first report of using blunt microcannulas for an enzymatic SMF reduction. The favorable results achieved with this innovative technique were validated through 3D-SQ. Prior studies have demonstrated the effectiveness of 3D imaging as a valuable tool for objectively assessing fat loss in the submental area following DAc injections (Li et al., 2018). It's important to note that this is a single case report; however, a comprehensive, long-term follow-up of this novel technique was implemented and objectively evaluated. Additionally, it would be essential to compare the results obtained with DAc to other established treatments in the literature, such as submental liposuction, which may be considered one of the most performed cosmetic procedure worldwide,^14^ high-intensity focused ultrasound, laser, cryolipolysis, among others.

In conclusion, this case report underscores the effectiveness of an innovative cannula-based approach for SMF reduction with DAc, assessed through 3D-SQ. Blunt microcannulas may represent a safer alternative to hypodermic needles, reducing the risk of severe complications. This approach holds promise for achieving facial and neck aesthetic harmony non-invasively, making it a valuable addition to minimally invasive aesthetic procedures.

## Funding

This study received no funding.

## Source of funding

This case report received no funding.

## Declaration of competing interest

The authors report no conflict of interests.
